# Efficacy of Traditional Anti-lipidemic Drugs in Lowering Lipoprotein(a) Levels: A Systematic Review

**DOI:** 10.7759/cureus.69824

**Published:** 2024-09-20

**Authors:** Mohit Sinha, Rafik Maged, Pakeeza Tarar, Venkata Varshitha Bandi, Hema Manvi Koneru, Hooria Sarwar

**Affiliations:** 1 Internal Medicine, Jawaharlal Nehru Medical College, Belgaum, IND; 2 Internal Medicine, Ain Shams University, Cairo, EGY; 3 Internal Medicine, Allama Iqbal Medical College, Lahore, PAK; 4 Internal Medicine, Guntur Medical College, Guntur, IND; 5 Internal Medicine, Rajiv Gandhi Institute of Medical Sciences, Adilabad, IND; 6 Psychiatry, Inside Out CURE Psychiatry LLC, Princeton, USA

**Keywords:** antisense oligonucleotides, apheresis therapy, ascvd, lipoprotein (a), lp(a), pcsk-9 inhibitor, small interfering rna, statins

## Abstract

Lipoprotein(a), or Lp(a), was identified in the early 1960. Its role as an independent risk factor for atherosclerotic cardiovascular disease (ASCVD) became widely recognized by the late 20th century, regardless of other traditional risk markers such as low-density lipoproteins and high-density lipoproteins.

This study aimed to systematically review available literature and compare the efficacy of different lipid-lowering drugs, both approved for clinical use and currently undergoing trials, in lowering Lp(a) levels.

A comprehensive search of medical databases including PubMed, PubMed Central (PMC), Medline, ScienceDirect, Cochrane Library, and Google Scholar was conducted to identify relevant studies. A total of 29 research papers met the inclusion criteria, focusing on the impact of various lipid-lowering drugs on Lp(a) concentration in patients with significantly elevated baseline Lp(a) levels.

Plasma Lp(a) levels exceeding 30 mg/dL are associated with a higher risk of ASCVD, including myocardial infarction, stroke, aortic valve stenosis, heart failure, peripheral arterial disease, and increased all-cause mortality. Most commonly used lipid-lowering agents, such as statins, fibrates, ezetimibe, and nutraceuticals like coenzyme Q10 (CoQ10), showed no significant effect on Lp(a) plasma levels. However, Lp(a) apheresis and proprotein convertase subtilisin/kexin type 9 (PCSK-9) inhibitors were found to effectively reduce plasma Lp(a) concentrations. Emerging therapies targeting apolipoprotein(a) RNA, including anti-sense oligonucleotides (ASO) and small interfering RNA (siRNA), significantly reduced Lp(a) levels in Phase 2 trials.

While several lipid-lowering agents have minimal impact on Lp(a) levels, therapies like Lp(a) apheresis, PCSK-9 inhibitors, and novel RNA-targeting drugs show promise in effectively reducing Lp(a) concentrations. However, whether these reductions translate into decreased cardiovascular events remains to be determined.

## Introduction and background

**Figure 1:**
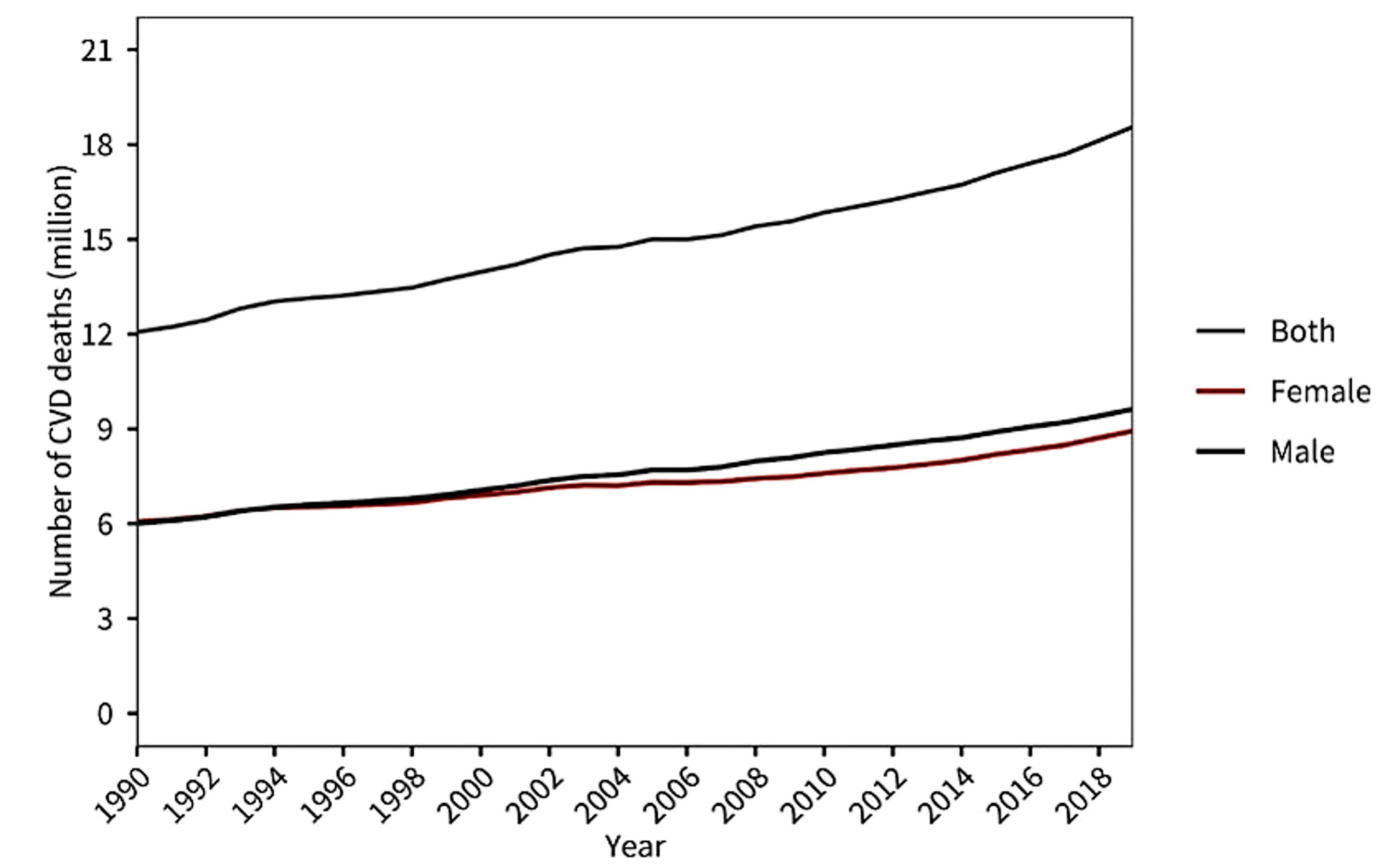
CVD as the primary cause of mortality from 1990 to 2019. Adapted from [1] CVD: Cardiovascular Disease

Atherosclerotic cardiovascular disease (ASCVD) refers to a group of conditions caused by the buildup of atherosclerotic plaques in the arteries, leading to narrowed or blocked blood vessels. This process affects various arteries, including those supplying the heart, brain, and other organs, leading to diseases such as coronary artery disease (CAD), stroke, and peripheral artery disease. Ischemic heart disease (IHD), also known as CAD, is a condition in which the blood flow to the heart muscle is reduced due to the narrowing or blockage of coronary arteries, usually caused by atherosclerosis (plaque buildup). ASCVD continues to affect more than half a billion people around the world. The leading cause of premature death in 146 countries for men and 98 countries for women is IHD. In 2021, ASCVD claimed the lives of 20.5 million people, a figure that accounted for around one-third of all global deaths and was a significant increase from the 12.1 million ASCVD deaths recorded in 1990 [1]. Figure 1 denotes the rising trend in mortality due to cardiovascular disease (CVD) from 1990 to 2019. 

It has been known that lipid disorders have been among the most common ASCVD risk factors in the world. Therefore, the assessment of the lipid profile plays a fundamental role in the analysis of ASCVD risk [2]. Traditionally, adequately lowering the serum concentration of low-density lipoprotein cholesterol (LDL-C) has been the primary goal of lipid-lowering treatment. Indeed, numerous randomized controlled trials have shown that every mmol/L reduction of plasma LDL-C level leads to a decrease in the risk of ASCVD by 20-25% after 5 years and 50-55% after 40 years [3].

However, despite effective reduction of LDL-C levels, many patients still remain at an elevated risk of ASCVD. This can be primarily attributed to lipoprotein(a) (Lp(a)), but also non high-density lipoprotein (HDL) cholesterol and triglycerides [4]. Increased serum Lp(a) concentration is an important risk factor for ASCVD because it is independent of the serum LDL-C [5]. In the Multi-Ethnic Study of Atherosclerosis (MESA) prospective study conducted by Rikhi et al., which comprised 4,585 participants and followed over the course of a median of 13.4 years, it was found that elevated Lp(a) level increased the risk of coronary heart disease (CHD) even when LDL-C was in the normal range. The MESA study also suggested that the group of participants who had an elevated LDL-C level but in whom the Lp(a) was < 50mg/dl did not suffer from an increased lifetime risk of CHD compared to the reference group. In summary, the MESA study concluded that there was an increased risk of CHD events when Lp(a) is elevated regardless of the baseline LDL-C (even when optimal) in the setting of primary prevention [6].

In the prospective investigation conducted by Afshar et al. involving 2606 Framingham Heart Study (FHS) participants, it was confirmed that both lipoprotein(a) ≥100 nmol/L and LDL‐C ≥135 mg/dL, were each significantly associated with an increased incidence of CVD over 15 years. Most importantly, it was shown that in individuals with only moderate elevations of LDL‐C (130-159 mg/dL), the presence of high lipoprotein(a) puts individuals at high risk, equivalent to that seen for individuals with LDL‐C ≥160 mg/dL, which according to recent lipid guidelines is a risk enhancer and a possible indication for earlier therapeutic intervention. Furthermore, they concluded that absolute cardiovascular event rates over a 15‐year period were highest among individuals with both high lipoprotein(a) (≥100 nmol/L) and LDL‐C ≥135 mg/dL, reaching up to 22.6% [5].

In a study conducted by Kaiser et al., it was found that in patients with multi-vessel CAD and receiving appropriate primary prevention therapy, high Lp(a) levels were associated with accelerated progression of low-attenuation plaques (necrotic core). This association between elevated Lp(a) and plaque progression and consequently increased risk of myocardial infarction supports the notion of targeting Lp(a) in preventing ASCVD events [7].

The normal range value of serum Lp(a) concentration has not been established yet. It is recommended that serum Lp(a) levels, whether measured in a fasting or non-fasting state, should be below 30 mg/dL. Based on the serum Lp(a) concentration, the cardiovascular risk can be determined: 30-50 mg/dL as moderate risk, more than 50 mg/dL as high risk and more than 180 mg/dL as very high cardiovascular risk [8].

As per American College of Cardiology/American Heart Association (ACC/AHA) guidelines and the Canadian Cardiovascular Society (CCS) guidelines, Lp(a) level anything more than 50 mg/dL should be targeted. Also, the European Atherosclerotic Society (EAS) consensus statement defines Lp(a) level less than 30 mg/dL as normal, 30-50 mg/dL as intermediate, and more than 50 mg/dL as abnormal [9].

In a study of 3359 patients from the Atherothrombosis Intervention in Metabolic Syndrome with low HDL/HIGH Triglycerides (AIM-HIGH) trial, hazard ratio (HR) for CVD events adjusted for age, gender, trial treatment, LDL-C, and other risk factors, it was found that the HR increased from 1.04 (0.82 to 1.32) to 1.51 (1.25 to 1.84) concurrently for Lp(a) level from 15 to 30 mg/dL up to more than 70 mg/dL [10].

Meta-analyses of 36 epidemiological studies including the Emerging Risk Factors collaboration showed that the risk ratio (RR) for CHD (adjusted for age and sex) was 1.16 (1.11-1.22) per 3.5-fold higher Lp(a) concentration. A plasma concentration of 20 mg/dL was associated with a 1.5-fold risk elevation while levels exceeding 50 mg/dl were associated with a 2-fold risk elevation [11].

There are currently no approved pharmacologic therapies that specifically target the Lp(a). The primary objective of this review is to learn about and investigate the impact of both approved and in-trial anti-lipidemic drugs in lowering the Lp(a) levels, either alone or in conjunction.

## Review

Methods

This review was conducted in accordance with the Preferred Reporting Items for Systematic Reviews and Meta-Analyses (PRISMA) guidelines [12].

Eligibility Criteria

**Figure 2:**
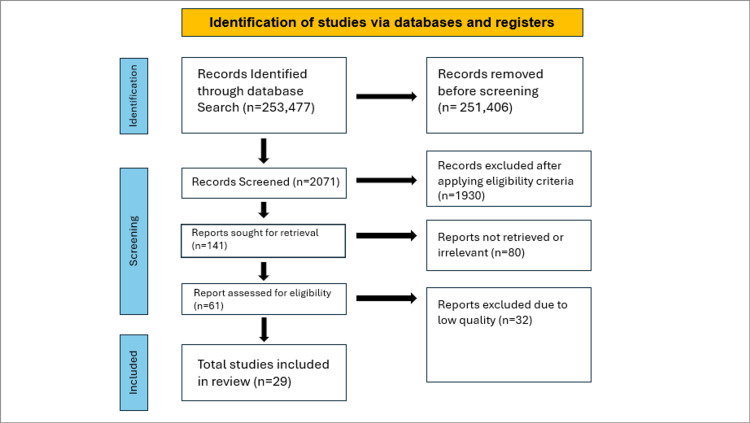
PRISMA flowchart describing the data collection and study selection processes. PRISMA: Preferred Reporting Items for Systematic Reviews and Meta-Analyses

Only studies meeting the following criteria were included: (1) research published between 1990 and 2024, (2) full-text articles, (3) studies involving human subjects only, and (4) studies with participants aged 18 years or older. We excluded animal studies, non-English articles, books, and documents. Additionally, research and review articles published before 1990 were excluded to focus on the most recent advancements in therapies. The process for selecting the literature is illustrated in the PRISMA flow diagram in Figure 2.

Search Strategy

This integrative review conducted a search for relevant articles indexed in PubMed, PubMed Central (PMC), Medline, Cochrane Library, Google Scholar, and Science Direct up to July 31, 2024. The search utilized Medical Subject Headings (MeSH) terms and keywords, including Lp(a), Lipoprotein a, Statin, Proprotein Convertase Subtilisin/Kexin type 9 (PCSK9), Inclisiran, Ezetimibe, Niacin, Fibrates, and Coenzyme Q10 (CoQ10). These terms were used both individually and in combination. The retrieved articles were then screened and filtered according to the predefined eligibility criteria.

Additionally, a combination of the aforementioned keywords and MeSH terms was employed to identify relevant records from the PubMed databases. The MeSH strategy used for searching included:

Lipoprotein(a) OR Lp(a) OR "Lipoprotein(a)"[Majr] AND ( "Lipoprotein(a)/adverse effects"[Majr] OR "Lipoprotein(a)/antagonists and inhibitors"[Majr] OR "Lipoprotein(a)/biosynthesis"[Majr] OR "Lipoprotein(a)/blood"[Majr] OR "Lipoprotein(a)/drug effects"[Majr] OR "Lipoprotein(a)/genetics"[Majr] OR "Lipoprotein(a)/metabolism"[Majr] OR "Lipoprotein(a)/pharmacokinetics"[Majr] OR "Lipoprotein(a)/pharmacology"[Majr] OR "Lipoprotein(a)/physiology"[Majr] OR "Lipoprotein(a)/poisoning"[Majr] OR "Lipoprotein(a)/toxicity"[Majr] ) AND statins OR Rosuvastatin OR Atorvastatin OR Simvastatin OR Lovastatin OR Pravastatin OR Fluvastatin OR Pitavastatin OR ( "Hydroxymethylglutaryl-CoA Reductase Inhibitors/administration and dosage"[Majr] OR "Hydroxymethylglutaryl-CoA Reductase Inhibitors/adverse effects"[Majr] OR "Hydroxymethylglutaryl-CoA Reductase Inhibitors/chemistry"[Majr] OR "Hydroxymethylglutaryl-CoA Reductase Inhibitors/pharmacokinetics"[Majr] OR "Hydroxymethylglutaryl-CoA Reductase Inhibitors/pharmacology"[Majr] OR "Hydroxymethylglutaryl-CoA Reductase Inhibitors/therapeutic use"[Majr] OR "Hydroxymethylglutaryl-CoA Reductase Inhibitors/toxicity"[Majr] ) AND PCSK9 inhibitors OR Evolocumab OR Alirocumab OR ( "PCSK9 Inhibitors/administration and dosage"[Majr] OR "PCSK9 Inhibitors/adverse effects"[Majr] OR "PCSK9 Inhibitors/blood"[Majr] OR "PCSK9 Inhibitors/metabolism"[Majr] OR "PCSK9 Inhibitors/pharmacokinetics"[Majr] OR "PCSK9 Inhibitors/pharmacology"[Majr] OR "PCSK9 Inhibitors/therapeutic use"[Majr] ) AND Fibrates OR Gemfibrozil OR Clofibrate OR Fenofibrate OR Bezafibrate OR Ciprofibrate OR ( "Fibric Acids/administration and dosage"[Majr] OR "Fibric Acids/adverse effects"[Majr] OR "Fibric Acids/agonists"[Majr] OR "Fibric Acids/antagonists and inhibitors"[Majr] OR "Fibric Acids/blood"[Majr] OR "Fibric Acids/metabolism"[Majr] OR "Fibric Acids/pharmacokinetics"[Majr] OR "Fibric Acids/pharmacology"[Majr] OR "Fibric Acids/therapeutic use"[Majr] ) AND CoQ10 OR ( "Ubiquinone/administration and dosage"[Majr] OR "Ubiquinone/adverse effects"[Majr] OR "Ubiquinone/analogs and derivatives"[Majr] OR "Ubiquinone/antagonists and inhibitors"[Majr] OR "Ubiquinone/biosynthesis"[Majr] OR "Ubiquinone/blood"[Majr] OR "Ubiquinone/deficiency"[Majr] OR "Ubiquinone/drug effects"[Majr] OR "Ubiquinone/metabolism"[Majr] OR "Ubiquinone/pharmacokinetics"[Majr] OR "Ubiquinone/pharmacology"[Majr] OR "Ubiquinone/physiology"[Majr] OR "Ubiquinone/therapeutic use"[Majr] ) AND Niacin OR ( "Niacin/administration and dosage"[Majr] OR "Niacin/adverse effects"[Majr] OR "Niacin/agonists"[Majr] OR "Niacin/analogs and derivatives"[Majr] OR "Niacin/antagonists and inhibitors"[Majr] OR "Niacin/blood"[Majr] OR "Niacin/isolation and purification"[Majr] OR "Niacin/metabolism"[Majr] OR "Niacin/pharmacokinetics"[Majr] OR "Niacin/pharmacology"[Majr] OR "Niacin/physiology"[Majr] OR "Niacin/therapeutic use"[Majr] OR "Niacin/toxicity"[Majr] )

Quality Appraisal of the Shortlisted Articles

For the shortlisted articles, the appropriate quality appraisal tools were applied according to the study type. Narrative reviews were assessed using the Scale for the Assessment of Narrative Reviews (SANRA) [13]. The Assessment of Multiple Systematic Reviews (AMSTAR 2) [14] tool was used for systematic reviews. Randomized controlled trials (RCTs) were evaluated with the Cochrane risk-of-bias tool (RoB 2) [15] for randomized trials while the Joanna Briggs Institute (JBI) [16] tool was employed to evaluate observational studies. Only those articles that met the quality appraisal standards were included in this systematic review. The primary outcome of interest was the reduction in plasma Lp(a) levels. Table 1 shows the quality appraisal results using SANRA.

**Table 1:** Quality appraisal using the SANRA tool. SANRA: Scale for the Assessment of Narrative Review

SANRA	Ference et al., 2018 [3]	Kamanna et al., 2008 [17]	Franchini et al., 2016 [18]	Malick et al., 2018 [19]
Justification of the article’s importance for the readership	2	2	2	2
Statement of concrete aims and formulation of questions	2	2	2	2
Description of the literature search	1	2	2	0
Referencing	1	2	2	2
Scientific reasoning	2	2	2	2
Appropriate presentation of data	2	2	2	2

Table 2 presents the quality appraisal results based on the AMSTAR 2 tool.

**Table 2:** Quality appraisal using the AMSTAR 2 tool. +: Yes; -: No; AMSTAR 2: Assessment of Multiple Systematic Review 2

AMSTAR 2	Willeit et al., 2018 [20]	Awad et al., 2018 [21]	Sahebkar et al., 2018 [22]	Sahebkar et al., 2017 [23]	Sahebkar et al., 2016 [24]	Jorat et al., 2018 [25]
Population, intervention, comparison, and outcome (PICO) components	+	+	+	+	+	+
Pre-established review methods and any substantial protocol deviation	+	_	_	_	_	_
Justification for selection of study designs	+	+	+	+	+	+
Search strategy for the literature explained	+	+	+	+	+	+
Duplicate study selection performed	+	+	+	+	+	+
Duplicate data extraction was performed	+	+	+	+	+	+
Justification for the excluded studies provided	+	+	+	+	+	+
Detailed description of the included studies	+	+	+	+	+	+
Assessment of the risk of bias (RoB) in individual studies	+	+	+	+	+	+
Reporting on the funding sources	+	+	+	+	+	+
Appropriate methods used for statistical combination of results	+	+	+	+	+	+
Impact of RoB in individual studies on the results of the meta-analysis	+	+	+	+	+	+
RoB used in interpreting the results	+	+	+	+	+	+
Explanation of heterogeneity in the results	+	+	+	+	+	+
Investigation of publication bias and its impact on the results	+	+	+	+	+	+
Conflict of interest and funding	+	+	+	+	+	+

Table 3 displays the quality appraisal results using the Cochrane RoB 2 tool.

**Table 3:** Quality assessment using the Cochrane RoB 2 tool +: Low risk; ?: Some concerns; -: High risk; Cochrane RoB: Risk-of-bias tool for randomized trials.

Cochrane RoB 2	Albers et al., 2013 [26]	Khera et al., 2014 [27]	Arsenault et al., 2014 [28]	Fraley et al., 2001 [29]	Choi et al., 2008 [30]	Cannon et al., 2015 [31]	Raal et al., 2016 [32]	Watts et al., 2018 [33]	Bittner et al., 2019 [34]	Boden et al., 2011 [35]	Landray et al., 2013 [36]	O’Donoghue et al., 2022 [37]	Tsimikas et al., 2020 [38]	Ray et al., 2020 [39]
Randomization process	+	+	+	+	+	+	+	+	+	+	+	+	+	+
Deviations from intended interventions	+	+	+	+	+	+	+	+	+	+	+	+	+	+
Missing outcome data	+	+	+	+	+	+	+	+	+	+	+	+	+	+
Measurement of the outcome	+	+	+	+	+	+	+	+	+	+	+	+	+	+
Selection of the reported result	+	+	+	+	+	+	+	+	+	+	+	+	+	+

Table 4 presents the results of the quality appraisal conducted with the JBI tool.

**Table 4:** Evaluation of quality using the JBI tool +: Yes; -: No; ?: Unclear; N/A: Not applicable; JBI: Joanna Briggs Institute

JBI	Wei et al., 2018 [40]	Capoulade et al., 2015 [41]	Roeseler et al., 2016 [42]	Safarova et al., 2013 [43]	Afshar et al., 2020 [5]	Rikhi et al., 2022 [6]	Kaiser et al., 2021 [7]	Wong et al., 2021 [10]
Was the study design appropriate for the research question?	+	+	+	+	+	+	+	+
Was the study conducted in an appropriate setting?	+	+	+	+	+	+	+	+
Were the participants selected appropriately?	+	+	+	+	+	+	+	+
Was the sample size adequate?	+	+	+	+	+	+	+	+
Were the outcomes measured appropriately?	+	+	+	+	+	+	+	+
Was there an appropriate statistical analysis?	+	+	+	+	+	+	+	+
Were the results valid and reliable?	+	+	+	+	+	+	+	+

Results

After searching the PubMed, PMC, Medline, Cochrane Library, Google Scholar, and Science Direct databases, a total of 253,477 records were identified. Following the removal of duplicates and irrelevant articles, 141 full-text articles were selected for eligibility screening. EndNote Basic (Clarivate, London, United Kingdom) was utilized as the reference manager for data analysis. Of these, 61 studies were initially pre-qualified for quality evaluation. Ultimately, 29 studies were included in this review. Table 5 provides a summary of the finalized articles.

**Table 5:** Summary of the finalized articles. ASCVD: Atherosclerotic Cardiovascular Disease; LDL-C: Low-Density Lipoprotein Cholesterol; HDL: High-Density Lipoprotein; Lp(a): Lipoprotein(a); LPA gene: Lipoprotein(a) gene; MESA: Multi-Ethnic Study of Atherosclerosis; TNT Trial: Treating to New Targets trial; MIRACL Trial: Myocardial Ischemia Reduction with Aggressive Cholesterol Lowering Trial; ACS: Acute Coronary Syndrome; REVERSAL: Reversal of Atherosclerosis with Aggressive Lipid LoweringPCSK9: Proprotein Convertase Subtilisin/Kexin type 9; siRNA: small interfering Ribonucleic Acid

References	Year of Study	Study Design	Participants	Results	Outcomes/Conclusion	Interpretation
Afshar et al. [5]	2020	Cohort Study	2,657 participants from the Framingham Heart Study.	Concomitant elevations in lipoprotein(a) and LDL-C were associated with a significantly higher risk of incident cardiovascular disease compared to elevations in LDL-C alone.	Elevated levels of both lipoprotein(a) and LDL-C increase the risk of cardiovascular disease more than elevated LDL-C alone. The study underscores the importance of considering both lipoprotein(a) and LDL-C in cardiovascular risk assessment.	The findings suggest that both lipoprotein(a) and LDL-C should be targeted for intervention in patients at risk of cardiovascular disease. This highlights the need for comprehensive lipid management strategies.
Rikhi et al. [6]	2022	Cross-Sectional Study	6,814 participants from the Multi-Ethnic Study of Atherosclerosis (MESA).	Elevated Lp(a) levels were significantly associated with an increased risk of ASCVD. Each standard deviation increase in Lp(a) was associated with a 25% higher risk of ASCVD.	Lp(a) is an important predictor of ASCVD risk in the general population. The study suggests incorporating Lp(a) measurement into routine risk assessments.	This research underscores Lp(a) as a significant risk factor for ASCVD in the general population and emphasizes its potential utility in preventive cardiology.
Kaiser et al. [7]	2022	Prospective Cohort Study	191 patients with atherosclerotic disease undergoing imaging.	Higher levels of lipoprotein(a) were associated with increased progression of atherosclerotic plaques.	Elevated lipoprotein(a) is linked to increased progression of atherosclerotic plaques, suggesting its role as a potential risk factor for plaque development.	The association between elevated lipoprotein(a) and plaque progression supports the need for further investigation into lipoprotein(a) as a target for therapeutic interventions to mitigate atherosclerotic disease.
Wong et al. [10]	2021	Retrospective cohort study	11,770 statin-treated adults with cardiovascular disease.	The study found that higher levels of lipoprotein(a) were associated with an increased risk of first and recurrent atherosclerotic cardiovascular disease events in statin-treated patients.	Elevated lipoprotein(a) levels were a significant predictor of recurrent cardiovascular events, suggesting that lipoprotein(a) may be an important marker for ongoing risk in statin-treated individuals.	Monitoring and potentially targeting lipoprotein(a) levels could be beneficial for risk stratification and management in patients with cardiovascular disease, even when they are on statin therapy.
Emerging Risk Factors Collaboration Group [11]	2009	Meta-analysis of prospective cohort studies	23,455 participants from 15 studies	Elevated lipoprotein(a) concentrations were associated with increased risks of coronary heart disease and stroke. The association with nonvascular mortality was less clear but indicated a possible increased risk.	Higher levels of lipoprotein(a) are a significant risk factor for coronary heart disease and stroke. The study supports the consideration of lipoprotein(a) as a potential marker for cardiovascular risk assessment.	Lipoprotein(a) could be a valuable biomarker for identifying individuals at higher risk for coronary heart disease and stroke, suggesting its utility in cardiovascular risk stratification.
Willeit et al. [20]	2018	Meta-analysis	18,226 participants across multiple statin trials	Baseline lipoprotein(a) levels were predictive of cardiovascular events in patients treated with statins. On-statin treatment lipoprotein(a) levels were also associated with cardiovascular outcomes.	Elevated baseline lipoprotein(a) levels are a significant predictor of cardiovascular events. The study supports monitoring lipoprotein(a) levels in patients undergoing statin therapy for better risk assessment and management.	Lipoprotein(a) levels can help in identifying patients at higher risk for cardiovascular events, potentially guiding more tailored treatment strategies in those on statin therapy.
Albers et al. [26]	2013	Randomized Controlled Trial	3,414 participants with atherothrombosis, low HDL cholesterol, and high triglycerides.	Higher levels of apolipoprotein B were associated with increased cardiovascular events, while apolipoprotein A-1 was associated with lower risk. Lipoprotein(a) levels did not show a significant association with cardiovascular outcomes in this trial.	Apolipoprotein B and A-1 levels are important markers for cardiovascular risk, whereas lipoprotein(a) did not demonstrate a significant predictive value in this study. The study highlights the role of apolipoproteins in cardiovascular risk assessment.	Monitoring apolipoprotein levels could be more informative for assessing cardiovascular risk than lipoprotein(a) levels in patients with metabolic syndrome and atherothrombosis.
Khera et al. [27]	2014	Randomized Controlled Trial	17,802 participants with elevated C-reactive protein levels and low-density lipoprotein (LDL) cholesterol levels.	Rosuvastatin therapy reduced LDL cholesterol levels and C-reactive protein but had a modest effect on reducing lipoprotein(a) levels. High lipoprotein(a) concentrations were associated with residual cardiovascular risk despite statin therapy.	The study found that even with effective LDL cholesterol reduction by rosuvastatin, high levels of lipoprotein(a) were associated with continued vascular risk, indicating that lipoprotein(a) might contribute to residual risk despite statin therapy.	Lipoprotein(a) remains a significant risk factor for cardiovascular events that is not fully addressed by current statin therapy. This underscores the need for additional strategies to manage residual vascular risk in patients on statins.
Wei et al. [40]	2018	Cohort Study	26,297 participants who were receiving statin therapy and had genetic data available.	The study identified specific genetic variants in the Lipoprotein(a) gene (LPA gene) that were associated with residual cardiovascular risk despite statin therapy. These variants were found to influence lipoprotein(a) levels and contribute to ongoing cardiovascular risk.	Genetic variants in Lipoprotein(a) gene (LPA gene) were associated with residual risk for cardiovascular events in patients on statins. This suggests that genetic predisposition to higher lipoprotein(a) levels may not be fully mitigated by statin therapy.	The findings highlight the role of genetic factors in determining residual cardiovascular risk and suggest that addressing genetic predispositions may be important for improving risk management in patients on statins.
Tsimikas et al. [44]	2019	Narrative review	Not applicable (review article)	The review discusses the role of lipoprotein(a) in cardiovascular risk, summarizing evidence from various studies. It highlights the association between elevated lipoprotein(a) levels and increased risk of cardiovascular diseases. The review also examines the potential mechanisms through which lipoprotein(a) contributes to atherosclerosis and cardiovascular events.	Elevated lipoprotein(a) is a significant risk factor for cardiovascular diseases, and its levels are not fully addressed by current lipid-lowering therapies. The review emphasizes the need for more research into targeted therapies for reducing lipoprotein(a) levels.	The findings suggest that lipoprotein(a) plays a crucial role in cardiovascular risk beyond traditional lipid measurements. Further studies are needed to develop specific treatments for managing lipoprotein(a) and reducing associated cardiovascular risks.
Arsenault et al. [28]	2014	Cohort Study	10,001 statin-treated stable coronary artery disease patients from the Treating to New Targets (TNT) trial	The study evaluated the predictive value of lipid and non-lipid biomarkers for cardiovascular events in patients treated with statins. It found that while traditional lipid measures (e.g., LDL-C) are important, non-lipid biomarkers also provided additional predictive value for cardiovascular outcomes.	Non-lipid biomarkers, in addition to traditional lipid measures, can enhance risk prediction for cardiovascular events in statin-treated patients. This suggests that incorporating a broader range of biomarkers may improve patient risk stratification and management.	The results indicate that using a combination of lipid and non-lipid biomarkers could potentially lead to better risk assessment and treatment strategies for patients with stable coronary artery disease on statins. Further studies are needed to confirm these findings and to evaluate the clinical utility of these additional biomarkers.
Fraley et al. [29]	2009	Cohort Study	3,065 patients with acute coronary syndromes (ACS) enrolled in the Myocardial Ischemia Reduction with Aggressive Cholesterol Lowering (MIRACL) trial	The study assessed the association of oxidized phospholipids and biomarkers of oxidized LDL with cardiovascular risk factors and inflammatory markers. It also evaluated the effect of statin therapy on these biomarkers. The results showed that higher levels of oxidized phospholipids were associated with increased cardiovascular risk and inflammation, and statin therapy significantly reduced these biomarkers.	Oxidized phospholipids and oxidized LDL biomarkers are linked to cardiovascular risk and inflammation in patients with acute coronary syndrome (ACS). Statin therapy effectively lowers these biomarkers, which could contribute to the reduction in cardiovascular events.	The study highlights the role of oxidized LDL and related biomarkers in cardiovascular risk and inflammation. It supports the use of statins not only for cholesterol lowering but also for their potential effects on reducing oxidative stress and inflammation, thereby potentially lowering cardiovascular risk. Further research could explore these biomarkers' role in predicting cardiovascular events and tailoring treatments.
Choi et al. [30]	2008	Cohort Study	345 patients with coronary artery disease enrolled in the Reversal of Atherosclerosis with Aggressive Lipid Lowering (REVERSAL) trial	The study evaluated the relationship between biomarkers of oxidized LDL, statin therapy, and changes in coronary atheroma volume as assessed by quantitative coronary angiography. It found that higher levels of oxidized LDL biomarkers were associated with greater atheroma volume, and aggressive statin therapy led to a reduction in both oxidized LDL levels and atheroma volume.	The study concluded that oxidized LDL biomarkers are related to the burden of atherosclerosis and that aggressive statin therapy can reduce both oxidized LDL levels and coronary atheroma volume, indicating a beneficial effect on atherosclerosis progression.	This study supports the role of oxidized LDL as a marker of atherosclerosis and demonstrates that intensive statin therapy not only lowers LDL cholesterol but also reduces oxidative stress and atheroma volume. This highlights the importance of targeting oxidized LDL in managing atherosclerosis and suggests that statin therapy has a significant impact on reducing atherosclerotic plaque.
Capoulade et al. [41]	2015	Prospective Cohort Study	258 patients with calcific aortic valve stenosis who were followed for a median of 2.6 years	The study investigated the role of oxidized phospholipids and lipoprotein(a) in the progression of calcific aortic valve stenosis. Elevated levels of oxidized phospholipids and lipoprotein(a) were found to be associated with faster progression of aortic stenosis. The study used echocardiographic measures to assess valve area and gradient changes over time.	The study concluded that both oxidized phospholipids and lipoprotein(a) are significant predictors of aortic stenosis progression. Elevated levels of these biomarkers were associated with more rapid disease progression, suggesting that they could be useful in identifying patients at higher risk for more severe disease progression.	The findings highlight the importance of oxidized phospholipids and lipoprotein(a) as potential biomarkers for monitoring the progression of aortic stenosis. These markers may be useful for stratifying patients based on their risk of rapid disease progression and could potentially inform treatment decisions and follow-up strategies.
Cannon et al. [31]	2015	RandomizedControlled Trial	18,144 patients with acute coronary syndrome (ACS) who were stabilized and receiving statin therapy	The study assessed the effect of adding ezetimibe to ongoing statin therapy in patients who had experienced an acute coronary syndrome. Ezetimibe, when added to statin therapy, further reduced low-density lipoprotein cholesterol (LDL-C) levels and demonstrated a significant reduction in major cardiovascular events, including myocardial infarction, stroke, and cardiovascular death.	The addition of ezetimibe to statin therapy led to a significant reduction in LDL-C and improved cardiovascular outcomes compared to statin therapy alone. The study concluded that ezetimibe is a valuable addition to statin therapy for further lowering LDL-C and reducing cardiovascular risk in patients with acute coronary syndrome.	This study supports the use of ezetimibe as an effective adjunctive therapy to statins for patients who have experienced an acute coronary syndrome. The findings suggest that more intensive lipid-lowering strategies can further reduce the risk of cardiovascular events in these high-risk patients.
Awad et al. [21]	2018	Systematic Review and Meta-Analysis of Randomized Controlled Trials	2337 patients with primary hypercholesterolemia, pooled from seven randomized controlled trials	The meta-analysis assessed the effect of ezetimibe monotherapy on plasma lipoprotein(a) (Lp(a)) levels. The study found that ezetimibe significantly reduced Lp(a) concentrations by 7.06% in patients with primary hypercholesterolemia compared to placebo.	Ezetimibe monotherapy was effective in lowering Lp(a) levels in patients with primary hypercholesterolemia. This suggests that ezetimibe can be a useful treatment option for managing elevated Lp(a) levels in addition to its lipid-lowering effects.	The findings support the use of ezetimibe as an effective monotherapy for reducing Lp(a) levels in patients with primary hypercholesterolemia. This has implications for managing patients with elevated Lp(a) who are at higher cardiovascular risk.
Sahebkar et al. [22]	2018	Systematic Review and Meta-Analysis of Randomized Controlled Trials	5188 subjects pooled from 10 randomized controlled trials.	The meta-analysis evaluated the impact of ezetimibe on plasma lipoprotein(a) (Lp(a)) levels, both as a monotherapy and in combination with statins. The study found that ezetimibe did not significantly reduced Lp(a) concentrations in both treatment scenarios compared to placebo or standard therapy.	Ezetimibe was not effective in lowering Lp(a) levels both when used alone and when combined with statins. This indicates that ezetimibe cannot be considered a valuable addition to lipid-lowering therapy, particularly for patients with elevated Lp(a) levels.	The results suggest that ezetimibe should not be considered for managing elevated Lp(a) levels, whether used alone or in combination with statins.
Sahebkar et al. [23]	2017	Systematic Review and Meta-Analysis of Head-to-Head Randomized Controlled Trials.	1388 patients from sixteen head-to-head randomized controlled trials comparing fibrates and statins	Fibrates were found to be more effective than statins in reducing plasma lipoprotein(a) concentrations.	Fibrates showed a greater reduction in plasma lipoprotein(a) levels compared to statins. This suggests that fibrates might be more effective in managing elevated Lp(a) levels.	Given the superior efficacy of fibrates over statins in lowering Lp(a) levels, statins should be preferred for patients with high Lp(a) concentrations. This information is useful for guiding lipid-lowering treatment strategies.
Raal et al. [32]	2016	Systematic Review and Meta-Analysis of Clinical Trials	3278 patients treated with evolocumab from 10 clinical trials	The study found that proprotein convertase subtilisin/kexin type 9 (PCSK9) inhibition with evolocumab led to significant reductions in lipoprotein(a) (Lp(a)) levels. The role of LDL receptors in this effect was also explored.	Evolocumab, a PCSK9 inhibitor, effectively reduces Lp(a) levels, and the reduction is linked to the activity of LDL receptors. This indicates that PCSK9 inhibition is a promising approach for managing high Lp(a) levels.	The findings support the use of PCSK9 inhibitors like evolocumab as an effective treatment for lowering Lp(a), which may be beneficial for patients with elevated Lp(a) and cardiovascular risk.
Watts et al. [33]	2018	Randomized Controlled Trial	63 healthy men with plasma apo(a) concentrations exceeding 5 nmol/L on evolocumab and atorvastatin.	The study demonstrated that evolocumab, a PCSK9 inhibitor, significantly affects the kinetics of Lp(a) particles, leading to reductions in Lp(a) levels and alterations in their dynamics.	PCSK9 inhibition with evolocumab leads to a reduction in Lp(a) levels and modifies the kinetics of Lp(a) particles, suggesting its potential benefits in managing elevated Lp(a) levels.	The study provides evidence that PCSK9 inhibitors like evolocumab can effectively alter Lp(a) particle kinetics and reduce Lp(a) levels, which may have implications for the treatment of patients with high Lp(a) and associated cardiovascular risk.
Bittner et al. [34]	2020	Randomized Controlled Trial	1,223 participants with acute coronary syndrome	Alirocumab treatment resulted in a significant reduction in Lp(a) levels. This reduction was associated with a lower incidence of cardiovascular events in patients post-acute coronary syndrome.	Alirocumab effectively lowers Lp(a) levels and reduces cardiovascular risk following an acute coronary syndrome. This supports its potential benefit in secondary prevention strategies.	The findings highlight the role of PCSK9 inhibitors, like Alirocumab, in managing residual cardiovascular risk through significant reductions in Lp(a) levels after acute coronary events.
Boden et al. [35]	2011	Randomized Controlled Trial	3,414 with low HDL cholesterol and on statin therapy	Niacin therapy did not significantly reduce the incidence of major cardiovascular events compared to placebo in patients already receiving intensive statin therapy. Niacin did, however, increase HDL cholesterol levels and lower triglycerides	The addition of niacin to intensive statin therapy did not provide additional cardiovascular benefit in reducing major cardiovascular events beyond what was achieved with statin therapy alone.	The study suggests that, despite improvements in lipid profiles with niacin, it does not confer further cardiovascular risk reduction in patients who are already on intensive statin treatment. This highlights the need for a reassessment of the role of niacin in contemporary lipid management strategies.
Landray et al. [36]	2014	Randomized Controlled Trial	25,673 high-risk patients (patients with cardiovascular disease or at high risk for cardiovascular events)	Extended-release niacin with laropiprant did not significantly reduce the risk of major cardiovascular events compared to placebo. There was also an increase in adverse effects, such as infections and gastrointestinal issues, associated with niacin use.	The study found no benefit of adding extended-release niacin with laropiprant to statin therapy in reducing cardiovascular events among high-risk patients. The risk of adverse effects with niacin therapy may outweigh potential benefits.	The results suggest that adding extended-release niacin to statin therapy does not improve cardiovascular outcomes in high-risk patients and may lead to increased side effects. This challenges the routine use of niacin for additional cardiovascular risk reduction in these patients.
Sahebkar et al. [24]	2016	Systematic Review and Meta-Analysis	271	Coenzyme Q10 supplementation was found to significantly reduce plasma lipoprotein(a) levels, but it did not significantly affect other lipid indices such as LDL-C, HDL-C, or triglycerides.	The study concluded that coenzyme Q10 supplementation effectively lowers plasma lipoprotein(a) levels, but does not have a significant impact on other lipid parameters.	This suggests that coenzyme Q10 could be considered as a targeted intervention for lowering lipoprotein(a) levels, but its role in improving overall lipid profiles or cardiovascular outcomes remains uncertain. Further studies are needed to evaluate its clinical significance and potential benefits.
Jorat et al. [25]	2018 Systematic Review and Meta-Analysis	Systematic Review and Meta-Analysis	678	Coenzyme Q10 supplementation was associated with significant improvements in total cholesterol, LDL-C, and HDL-C levels among patients with coronary artery disease. However, no significant effect was observed on triglyceride levels.	The study concluded that coenzyme Q10 supplementation may be beneficial in improving certain lipid profiles in patients with coronary artery disease, particularly in reducing total cholesterol and LDL-C levels while increasing HDL-C levels.	Coenzyme Q10 could be a useful adjunctive treatment for managing lipid profiles in coronary artery disease patients. Nonetheless, the clinical significance of these improvements and their impact on cardiovascular outcomes need further investigation.
Roeseler et al. [42]	2016	Prospective Cohort Study	50 patients with lipoprotein(a)-associated cardiovascular disease who underwent lipoprotein apheresis.	Lipoprotein apheresis led to significant reductions in lipoprotein(a) levels, and over 5 years, patients demonstrated a stabilization of cardiovascular events and improved outcomes. Apolipoprotein(a) characterization revealed various isoforms and their impact on treatment efficacy.	The study concluded that lipoprotein apheresis is an effective treatment for managing lipoprotein(a)-associated cardiovascular disease, providing long-term benefits in reducing lipoprotein(a) levels and stabilizing cardiovascular outcomes.	Lipoprotein apheresis can be a valuable therapeutic option for patients with high lipoprotein(a) levels and associated cardiovascular risks, contributing to improved clinical outcomes over extended follow-up periods.
Safarova et al. [43]	2013	Prospective Cohort Study	24 patients with coronary atherosclerosis who underwent specific lipoprotein(a) apheresis.	The study found that specific lipoprotein(a) apheresis resulted in significant regression of coronary atherosclerosis as assessed by quantitative coronary angiography. There was a notable reduction in the severity of atherosclerotic plaques and improvement in coronary artery lumen diameter.	Specific lipoprotein(a) apheresis is effective in promoting regression of coronary atherosclerosis and improving coronary artery morphology, as evidenced by quantitative coronary angiography.	The results support the use of lipoprotein(a) apheresis as a therapeutic approach to reduce coronary atherosclerosis in patients with elevated lipoprotein(a) levels. This treatment may be beneficial in managing coronary artery disease and improving patient outcomes.
O’Donoghue et al. [37]	2022	Randomized Controlled Trial	284 patients with cardiovascular disease and elevated lipoprotein(a) levels.	The study demonstrated that small interfering RNA (siRNA) therapy effectively reduced lipoprotein(a) levels in patients. The treatment was associated with a significant decrease in lipoprotein(a) concentrations compared to the placebo group. However, the impact on cardiovascular outcomes was still under evaluation.	Small interfering RNA therapy shows promise in reducing lipoprotein(a) levels, which may have implications for cardiovascular risk management. Further research is needed to determine the long-term effects on cardiovascular outcomes.	siRNA therapy represents a novel approach to lowering lipoprotein(a) and may become a valuable tool in managing patients with elevated lipoprotein(a) levels and associated cardiovascular risk. The study supports the potential of targeting lipoprotein(a) through genetic therapies as part of cardiovascular disease treatment strategies.
Tsimikas et al. [38]	2020	Randomized Controlled Trial	286 patients with cardiovascular disease and elevated lipoprotein(a) levels.	The study evaluated the efficacy of a novel therapy for reducing lipoprotein(a) levels in individuals with cardiovascular disease. The therapy led to a significant reduction in lipoprotein(a) levels compared to the placebo. However, the impact on cardiovascular events was not the primary endpoint of the study.	Reduction in lipoprotein(a) levels was achieved with the therapy, indicating potential for this treatment to lower cardiovascular risk associated with elevated lipoprotein(a). Further studies are needed to confirm the long-term benefits and effects on cardiovascular outcomes.	The study suggests that targeting lipoprotein(a) can be a viable strategy for managing patients with cardiovascular disease. The significant reduction in lipoprotein(a) highlights the potential for this therapy in cardiovascular risk reduction, although additional research is required to fully understand its impact on cardiovascular events.
Ray et al. [39]	2020	Randomized Controlled Trial	3,600 patients with elevated LDL cholesterol and a history of cardiovascular disease or high cardiovascular risk.	Inclisiran, a small interfering RNA (siRNA) drug, significantly reduced LDL cholesterol levels compared to placebo. The drug demonstrated sustained efficacy in lowering LDL cholesterol over a period of 18 months.	Inclisiran was effective in reducing LDL cholesterol and was well-tolerated. The results support its use as an addition to current lipid-lowering therapies for patients with elevated LDL cholesterol.	Inclisiran offers a promising option for managing high LDL cholesterol, with potential benefits for reducing cardiovascular risk. The sustained reduction in LDL cholesterol highlights its efficacy as a treatment modality. Further studies may evaluate its long-term cardiovascular benefits and safety profile.

Discussion

Statins

Statins are widely recognized as essential in both primary and secondary prevention of CVD, primarily due to their strong ability to lower LDL cholesterol. It was widely believed that lowering the LDL-C level below 1.8 mmol/L (70 mg/dL) through statins either alone or in combination with other drugs would also negate the risks associated with elevated Lp(a) levels. However, this belief has been refuted by evidence showing that high plasma Lp(a) remains a significant independent risk factor for CVD irrespective of the LDL levels. This means that even with optimal LDL-C levels, elevated Lp(a) continues to contribute to increased atherosclerosis and CVD risk [20,26].

Statin therapy generally does not lower plasma Lp(a) levels; in fact, many studies indicate that it can even increase them by up to 25%. This effect might be due to the fact that LDL receptors (LDLRs) do not significantly influence Lp(a) clearance [44].

Despite extensive clinical research, the impact of statins on Lp(a) levels remains uncertain. Recent studies, including post hoc analyses of the Treating to New Targets (TNT) [28] and Justification for the Use of Statins in Prevention: An Intervention Trial Evaluating Rosuvastatin (JUPITER) trials [27], suggest that statins have little to no effect on Lp(a) levels. Other trials, such as Myocardial Ischemia Reduction with Aggressive Cholesterol Lowering (MIRACL) [29], Reversal of Atherosclerosis with Aggressive Lipid Lowering (REVERSAL) [30], and Aortic Stenosis Progression Observation: Measuring Effects of Rosuvastatin (ASTRONOMER) [23], even report increases in Lp(a) levels. Overall, there is no conclusive evidence that statin therapy effectively lowers Lp(a) levels, indicating that LDLRs may not play a significant role in Lp(a) clearance.

Ezetimibe

Ezetimibe works by inhibiting cholesterol absorption at the brush border of the small intestine through the sterol transporter Niemann-Pick C1-Like1 (NPC1L1). This action decreases the amount of cholesterol delivered to the liver, lowers hepatic cholesterol stores, and enhances cholesterol clearance from the bloodstream. Ezetimibe demonstrates modest efficacy in lowering LDL-C, which was linked to significant cardiovascular benefits in the Improved Reduction of Outcomes: Vytorin Efficacy International trial (IMPROVE-IT) [31]. Given its ability to reduce plasma LDL-C levels and the structural similarities between Lp(a) and LDL particles, it is reasonable to hypothesize that ezetimibe might also affect plasma Lp(a) levels.

A meta-analysis of seven RCTs comprising 2337 patients conducted by Awad et al., concluded that ezetimibe monotherapy (10 mg/day) resulted in a modest 7.06% reduction in plasma Lp(a) levels in patients with primary hypercholesterolemia, which was statistically significant. However, according to current literature, this level of reduction is considered to have minimal clinical relevance [21].

On the other hand, in a meta-analysis of 10 studies comprising 15 treatment arms conducted by Sahebkar et al., it was found that ezetimibe therapy did not lead to a significant reduction of plasma Lp(a) levels, when used either alone or in conjunction with statins. This study concluded that ezetimibe’s therapeutic effect in CVD prevention was not due to Lp(a) lowering effect [22].

Fibrates

In a meta-analysis conducted by Sahebkar et al., it was concluded that fibrates are notably more effective than statins in lowering plasma Lp(a) concentrations [23]. Moreover, combining fibrates with statins can further enhance the reduction of Lp(a) levels achieved by statin therapy alone. Therefore, using fibrates in conjunction with statins may offer additional benefits in reducing cardiovascular disease risk, particularly for patients with elevated Lp(a) levels, by decreasing apo(a) expression and improving Lp(a) clearance [29].

Proprotein Convertase Subtilin/Kexin Type 9 (PCSK-9) Inhibitors

Anti-PCSK9 antibodies, such as evolocumab and alirocumab, not only significantly reduce plasma LDL-C and prevent major ASCVD events but also decrease Lp(a) levels by up to nearly 30%. In patients with a higher baseline Lp(a), their effects were even greater [32]. The precise mechanism through which PCSK9 inhibitors lower plasma Lp(a) remains unclear. It has been hypothesized that Lp(a) may have a lower affinity for LDLRs compared to LDL particles, and reducing LDL levels might decrease competition for LDLRs, potentially enhancing Lp(a) clearance. Additionally, it has been proposed that PCSK9 inhibitors alone reduce plasma Lp(a) by decreasing its production, while their combination with statins further lowers Lp(a) by increasing its catabolism [33].

However, conclusive evidence is still missing regarding whether PCSK9 inhibitors reduce ASCVD risk specifically through Lp(a) reduction, or if the observed benefits are primarily due to achieving much lower LDL-C levels. To achieve a meaningful additional reduction in the risk of major adverse cardiovascular events (MACE) through targeting Lp(a), it may be necessary to achieve greater reductions in Lp(a) levels than those currently possible with PCSK9 inhibitors [34]. This could potentially be realized through more potent therapies or in patients with very high baseline Lp(a) and LDL-C levels.

Niacin

In addition to its well-documented effects on increasing HDL-C and lowering LDL-C and triglycerides (TG), niacin also reduces plasma levels of Lp(a). The mechanisms behind this Lp(a) reduction may include decreased transcription of apo(a) [45] and reduced secretion of apoB, potentially through the inhibition of triglyceride synthesis [17].

In the Atherothrombosis Intervention in Metabolic Syndrome with Low HDL/High Triglycerides: Impact on Global Health Outcomes (AIM-HIGH) trial, the effects of extended-release niacin were evaluated in 3,414 patients with stable atherosclerotic disease, low baseline HDL-C, and elevated TG levels, all of whom were on background statin therapy. The study found that niacin treatment led to a 21% reduction in Lp(a) levels compared to placebo [35].

The larger Heart Protection Study 2-Treatment of HDL to Reduce the Incidence of Vascular Events (HPS2-THRIVE) trial took a similar approach, enrolling 25,773 high-risk patients with a history of CVD. Participants were randomized to receive either extended-release niacin with laropiprant or a placebo, in addition to statin therapy with or without ezetimibe. In the HPS2-THRIVE trial, Lp(a) levels were measured at one year in a randomly selected subset of 1,999 participants, with baseline levels not being available. The results showed a reduction in Lp(a) from 60.3 nmol/L at baseline to 50.7 nmol/L at one year, reflecting a 17.8% decrease [36]. This reduction was consistent with the findings from the AIM-HIGH trial.

Despite both studies demonstrating a potential benefit in lipoprotein levels, including an average 20% reduction in Lp(a) levels, neither study showed a reduction in cardiovascular event rates.

Coenzyme Q10 (CoQ10)

CoQ10 is an intracellular antioxidant that helps prevent cellular aging and dysfunction caused by oxidative stress. It is frequently used in the treatment of cardiomyopathy, and supplementation with CoQ10 has been shown to significantly improve heart function. Additionally, CoQ10 deficiency, which often occurs with aging, has been linked to an increased risk of type 2 diabetes mellitus (T2DM) and CVD.

In a meta-analysis of seven randomized controlled trials comprising a total of 409 subjects and conducted by Sahebkar et al., CoQ10 was found to cause a small but significant reduction in plasma Lp(a) levels. CoQ10 supplementation significantly decreased Lp(a) levels among patients with obesity, T2DM, and CVD, mainly in those with Lp(a) more than 30 mg/dL. Reduction of Lp(a) levels was inversely associated with the administered CoQ10 dose [24].

Another meta-analysis carried out by Jorat et al., comprising eight trials and 526 subjects, found no significant impact of CoQ10 supplementation on CoQ10 levels [25].

Lp(a) Apheresis

Lipoprotein apheresis (LA) is an effective treatment for lowering Lp(a) concentrations in patients with severe, progressive ASCVD and very high Lp(a) levels. This procedure involves physically removing lipoproteins from the blood, resulting in a significant reduction in Lp(a) of over 60% per treatment, with average long-term reductions around 30%. LA is typically used for patients with extremely high hypercholesterolemia who have not achieved adequate plasma lipoprotein levels despite rigorous lifestyle changes and the most intensive pharmacologic lipid-lowering therapies. However, LA is an invasive, often lifelong therapeutic method that involves several potential complications. Problems may arise from venous punctures, hypotensive episodes, and bleeding risks due to the anticoagulation required during LA sessions. Additionally, LA is costly and may be impractical for many patients, with its feasibility largely dependent on the healthcare reimbursement system. Despite these challenges, LA remains a crucial treatment option for managing patients with homozygous familial hypercholesterolemia, as well as for those with severe drug-resistant dyslipidemias and established CVD.

Early systematic reviews indicated that apheresis could reduce CVD events by 54-90% [18]. In a later study involving 154 patients with a baseline Lp(a) level of 108 mg/dL, apheresis achieved a 68% reduction in Lp(a) and an 81% reduction in CVD events [40]. One specific variant of apheresis, Lipopac, targets Lp(a) exclusively, though data on this approach are limited. In a study of 15 patients, Lipopac apheresis reduced Lp(a) levels by 75% and demonstrated angiographic benefits [41]. Table 6 summarizes the average impact of various anti-lipidemic drugs on plasma Lp(a) levels.

**Table 6:** Summary of the average impact of anti-lipidemic drugs on plasma Lp(a) levels. LDLR: Low-Density Lipoprotein Receptor; apo(a): Apolipoprotein (a); PCSK-9: Proprotein Convertase Subtilin/Kexin type 9; CoQ10: Coenzyme Q10; Lp(a): Lipoprotein (a)

Treatment	% reduction in Lp(a)	Mechanism of action	References
Statin	-5% to +20%	HMG-CoA inhibition and increased expression of low-density lipoprotein receptor (LDLR)	[20,23,26-30,44]
Ezetimibe	0 to -10%	Decreased synthesis of Lp(a) and increased uptake through LDLR	[21,22,31]
Fibrates	0 to -20%	Inhibition of apolipoprotein (a) (apo(a)) transcription	[29]
PCSK-9 Inhibitors	-25%	Increased removal by LDLR	[32-34]
Niacin	-20%	Decreased production of apo(a)	[17,35,36,45]
CoQ10	0-15%	Suppression of oxidative stress and increased production of HDL	[24,25]
Lp(a) Apheresis	60% (per procedure); 30% (long term)	Physical removal of Lp(a) particles	[18,40,41]

From the data above, it can be concluded that current lipid-lowering medications are generally ineffective in significantly reducing Lp(a) levels, with the exception of PCSK9 inhibitors. Statins tend to either slightly increase Lp(a) levels or have no significant effect. Ezetimibe has been reported to reduce Lp(a) levels by 7.6% according to one meta-analysis, though other studies have shown no change. Fibrates have shown mixed results regarding their impact on Lp(a), with some studies indicating a decrease and others showing no effect. Niacin can decrease Lp(a) levels by 23%, but it is not recommended due to its lack of proven mortality and morbidity benefits in cardiovascular disease and its adverse effect profile. Both Lp(a) apheresis and PCSK9 inhibitors can reduce plasma Lp(a) levels by approximately 20%-30% on average, alongside a much more significant reduction in LDL-C, with reductions of up to 70%. This substantial reduction in LDL-C complicates the assessment of the true impact of lowered Lp(a) on cardiovascular events.

Given widely available evidence that Lp(a) is an independent risk factor for ASCVD, drugs which can specifically lower Lp(a) in patients with an elevated baseline of Lp(a) and which also result in better CV outcomes are the need of the hour.

Currently new therapies targeting RNA, including antisense oligonucleotides (ASOs) and small interfering RNA (siRNA) aimed at apolipoprotein(a) - the principal protein component of Lp(a) - are currently undergoing pahse 2 and 3 trials (Table 2). These therapies have the potential to reduce Lp(a) concentrations by up to 90%. Because they specifically lower Lp(a) without affecting other lipoproteins, they may provide crucial insights into whether a reduction in Lp(a) also leads to a decrease in cardiovascular events, potentially completing the final piece of the puzzle in understanding Lp(a)'s role in cardiovascular risk.

Table 7 summarizes some of the promising trials using novel drugs which act through ASOs and siRNA and are undergoing investigation [19,37,38,39].

**Table 7:** Summary of the novel drug trials which act through siRNA and ASO and are under investigation. Lp(a): Lipoprotein(a); OCEAN(a) DOSE: Olpasiran trials of Cardiovascular Events And LipoproteiN[a] reduction–DOSE Finding Study; Apo(a): Apolipoprotein(a); HORIZON: Hyperlipoproteinemia(a) Randomized Intervention Study of Drug Targeting Lp(a) and Cardiovascular Events; MACE: Major Adverse Cardiovascular Event; ASCVD: Atherosclerotic Cardiovascular Disease; CVD: Cardiovascular Disease

Study Name, Phase (Therapy)	Mechanism	Population	Outcome	Reference
Olpasiran trials of cardiovascular events and lipoprotein[a] Reduction–DOSE Finding Study (OCEAN(a) DOSE), phase 2	small interfering RNA (siRNA) mediated reduction of Lp(a) synthesis in the liver	Participants 281 Age 18-80 years Lp(a) level more than 150 nmol/L Evidence of atherosclerotic cardiovascular disease (ASCVD)	Preliminary results indicated a reduction of 70.5% to 110.5% in Lp(a) levels.	[37]
Pelacarsen Akcea-Apolipoprotein(a)-LRx, phase 2	antisense oligonucleotide (ASO) against Apolipoprotein(a) (Apo(a))	Participants 286 18-100 years of age Lp(a) level more than mg/dL Diagnosed cardio-vascular disease (CVD) Must be on standard-of-care preventive therapy for CVD risk factors other than elevated Lp(a) levels	Preliminary results indicated a dose-dependent reduction of Lp(a) levels ranging from 35% to 80%.	[38]
Pelacarsen, Hyperlipoproteinemia(a) randomized intervention study of drug targeting Lp(a) and cardiovascular events (HORIZON), phase 3	antisense oligonucleotide (ASO) against Apo(a)	Participants 8323 Age 18-90 years Lp(a) level more than 70 mg/dL Established CVD	Incidence of major adverse cardiovascular event (MACE) in 4 years.	[19]
Inclisiran, Orion-11, phase 3	siRNA mediated inhibition of PCSK9 synthesis	≥18 years of age LDL-C more than 70 mg/dL History of ASCVD	Preliminary results indicated a reduction of Lp(a) level by 28.5%.	[39]

Limitations

Despite the comprehensive nature of this systematic review, several limitations must be acknowledged.

Variability in study designs included in this review, patient populations, drug dosages, and measurement techniques can lead to inconsistent results among different studies. This variability complicates the ability to draw definitive conclusions.

Many studies reviewed here have short follow-up periods, which did not capture the long-term effects of lipid-lowering drugs on Lp(a) levels and cardiovascular outcomes.

Also publication bias cannot be ruled out. Studies showing significant effects are more likely to be published than those with null or negative results, leading to potential overestimation of the impact of lipid-lowering drugs on Lp(a) levels.

Many studies focus solely on changes in Lp(a) levels rather than on clinical outcomes such as cardiovascular events, which limits the ability to assess the clinical significance of Lp(a) reduction.

## Conclusions

Numerous preclinical and clinical studies have underscored the significant role of elevated plasma Lp(a) in increasing the risk of ASCVD, independent of LDL-C levels. This highlights hyperlipoproteinemia (a) as a crucial target for ASCVD prevention. However, current evidence supporting Lp(a) reduction as a definitive and beneficial strategy for preventing ASCVD events is limited. Existing treatments for lowering plasma Lp(a) are not yet optimal, either due to their modest efficacy, lack of outcome data, or safety concerns. Promising new interventions and targeted therapies are under investigation in ongoing trials. Among these, ASO therapies targeting apolipoprotein(a) show early promise with encouraging results in terms of efficacy and safety. The next crucial step is to demonstrate that lowering Lp(a) translates into cardiovascular benefits for patients with high Lp(a) levels.
